# Supplementary material for: Livestock abortion surveillance in Tanzania reveals disease priorities and importance of timely collection of vaginal swab samples for attribution
Source: eLife. 2024 Dec 16;13:RP95296. doi: 10.7554/eLife.95296 (PMC11649233; doi:10.7554/eLife.95296)
Supplement: Supplementary file 1. [file elife-95296-supp1.docx]

**Supplementary File 1:**

**Herd data**

The mean (median, range) number of cattle, goats and sheep per herd were 29.8 (7, 0 – 276), 55.9 (24, 0 – 817) and 47.5 (20, 0 – 1000), respectively, giving an approximate median herd composition ratio of 1 cattle to 3 goats to 3 sheep. The mean (median and range) number of adult female cattle, goats and sheep per herd were 17.5 9 (4 and 0 – 164), 35.4 (15 and 0 – 409) and 32.0 (12, and 0 – 800), respectively. The mean percentage of adult female cattle, goats and sheep per herd was 63.1%, 62.6% and 66.1%, respectively.
