# Supplementary material for: Livestock abortion surveillance in Tanzania reveals disease priorities and importance of timely collection of vaginal swab samples for attribution
Source: eLife. 2024 Dec 16;13:RP95296. doi: 10.7554/eLife.95296 (PMC11649233; doi:10.7554/eLife.95296)
Supplement: Supplementary file 2. [file elife-95296-supp2.docx]

**Supplementary File 2:**

**Expected number of abortions per breed**

| **SPECIES** | **BREED** | **ACTUAL** | **EXPECTED** |
| --- | --- | --- | --- |
| Cattle | local | 16 | 61 |
| Goat | local | 77 | 94 |
| Sheep | local | 41 | 42 |
| Cattle | cross | 36 | 7 |
| Goat | cross | 17 | 2 |
| Sheep | cross | 3 | 2 |
| Cattle | exotic | 17 | 1 |
| Goat | exotic | 3 | 1 |
| Sheep | exotic | 0 | 0 |

The actual number of abortions reported for each species and breed and, based on the proportion of each breed in all the herds that reported cases, the expected number of abortions was calculated.
