# Supplementary material for: Livestock abortion surveillance in Tanzania reveals disease priorities and importance of timely collection of vaginal swab samples for attribution
Source: eLife. 2024 Dec 16;13:RP95296. doi: 10.7554/eLife.95296 (PMC11649233; doi:10.7554/eLife.95296)
Supplement: Supplementary file 3. [file elife-95296-supp3.docx]

**Supplementary File 3**

**SEBI Household Questionnaire [Dodoso la kaya]**

Enter household ID [Ingiza utambulisho wa kaya] [ ]

Language [Lugha]

🞎 Kiswahili

🞎 English

🞎 Maa

Interviewer ID [Utambulisho wa mhojiwa] [ ]

Name of village in which household is located [Jina la kijiji] [ ]

**1. RESPONDENT/HOUSEHOLD HEAD DETAILS**

1.1. How many different households are in this compound [Kuna kaya ngapi katika boma hili]? [ ]

1.2 What is the relationship of the respondent to the head of the compound [Nini uhusiano wa mhojiwa na mkuu wa kaya]?

🞎 Spouse [Mwenza]

🞎 Parent [Mzazi]

🞎 Child [Mtoto]

🞎 Sibling [Dada/kaka]

🞎 Self (= H of H) [Mimi mwenyewe]

🞎 Other [Nyingine] [ ]

1.3 What is the head of compound's gender [Jinsia ya mkuu wa kaya]?

🞎 Male [Mme] 🞎 Female [Mke]

1.4 What is the head of compound's year of birth [Mwaka wako wa kuzaliwa ni upi]? [ ]

1.5 What is the head of compound's age class [Upo kwenye kundi gani la umri]?

🞎 13-18

🞎 19-34

🞎 35-54

🞎 >55

1.6 What is the head of compound's tribe [Kabila lako ni lipi]?

🞎 Arusha

🞎 Barabaig

🞎 Chagga

🞎 Iraqw

🞎 Pare

🞎 Maasai

🞎 Sambaa

🞎 Other [ ]

1.7 What is the head of compound's highest level of education [Elimu yako cha juu ni ipi]?

🞎 No formal education

🞎 Some primary school

🞎 Completed primary school

🞎 Some secondary school

🞎 Completed secondary school

🞎 Post secondary qualifications

🞎 Some university

🞎 University completed

🞎 Post graduate

**2. COMPOUND/HOUSEHOLD DETAILS**

2.1 How many members of this compound are in the following age-groups [Wakazi wangapi wa boma hili wako katika kila kundi la kiumri]?

< 5 years [chini miaka 5] [ ]

5-18 years [miaka 5-18] [ ]

> 18 years [zaidi ya miaka 18] [ ]

2.2 How many inhabited buildings are there in this compound [Kuna majengo mangapi ya kuishi katika kaya yako]? [ ]

2.3 What sources of drinking water does your compound use during the wet or dry season [Ni chanzo kipi cha maji kaya yako huwa wanatumia wakati wa masika]?

🞎 Piped water into the home [Yanayosukumwa kwa bomba mpaka ndani nyumbani]

🞎 Public/communal well or standpipe [Kisima au pampu ya jumuia]

🞎 River or creek (moving water) directly [Moja kwa moja kutoka mto au mfereji (maji yanatotembea)]

🞎 Lake, pond, dam (standing water) directly [Moja kwa moja kutoka Ziwa, dimbwi bwawa (maji yaliyosimama)]

🞎 Private well or pump [Kisima au pampu ya binafsi]

🞎 From a spring [Kutoka katika chemchem]

🞎 Rainwater [Maji ya mvua]

🞎 Tanker truck [Tanki la gari]

🞎 Cart of wheelbarrow with small tank or drum [Mkokoteni na tanki dogo au madumu/pipa]

🞎 Bottled water [Maji ya chupa]

🞎 Other [ ] [Vyanzo vinginevyo]

2.4 How frequently do members of this compound treat water before drinking it [Huwa unatibu maji ya kunywa mara nyingi kabla ya kunywa]?

🞎 Always (daily)

🞎 Sometimes (a few times a week)

🞎 Rarely (a few times a month)

🞎 Never

2.5 How do you treat it [Ni mara ngapi huwa unatibu maji ya kunywa kabla ya kunywa]?

🞎 Boiling [Kuchemsha]

🞎 Strain it through a cloth [Kuchuja kwa nguo]

🞎 Adding disinfectant, such as chlorine or bleach [Kuweka dawa kama shabu/klorine]

🞎 Sedimentation and decant [Kuacha kwa muda yatwae/uchafu uende chini]

🞎 Filtering [Kuchujwa]

🞎 Solar disinfection [Kufanya salama kwa jua]

🞎 Other [Nyinginezo] [ ]

2.6 What type of toilet system do members of this compound use [Ni aina gani ya mfumo wa choo ambao unatumika kawaida na kaya yako]?

🞎 Flush or pour toilet with septic tank, including squat toilet [Choo cha kuvuta au cha kumwaga maji cha kuchuchumaa na mfumo wa shimo la maji taka]

🞎 Flush or pour toilet connected to sewer pipe, including squat toilet [Choo cha maji kilichounganishwa na bomba la maji taka, pamoja na choo cha kuchuchumaa]

🞎 Pit latrine with covering slab [Choo cha shimo kilichosakafiwa]

🞎 Pit latrine without covering slab [Choo cha shimo bila kusakafiwa]

🞎 Ventilated improved pit latrine (VIP) [Choo cha shimo bora chenya bomba la kutoa hewa chafu]

🞎 Bucket or plastic bags [Ndoo au mifuko ya plastiki/Rambo]

🞎 No facilities or field or bush [Hakuna choo, kwenda porini]

🞎 Other [ ]

2.7 What kind of electricity do you have in this compound [Je, una umeme wa aina gani katika kaya hii]?

🞎 Grid

🞎 Solar

🞎 Generator

🞎 Other

🞎 No electricity

🞎 Other [ ]

2.8 What energy sources are used for cooking in this compound [Aina gani nishati inatumika kwa kupikia katika kaya hii]?

🞎 Electricity [Umeme]

🞎 Gas [Gesi]

🞎 Kerosene [Kuni]

🞎 Cow dung [Kinyesi cha ng’ombe]

🞎 Firewood [Mafuta taa]

🞎 Charcoal [Mkaa]

🞎 Other [Nyinginezo] [ ]

2.9 For the house that the head of the compound sleeps in, what is the roof made of [Kwa nyumba anayolala mkuu wa kaya, paa limeezekwa na nini]?

🞎 Metal [Chuma/mabati]

🞎 Thatch [Nyasi]

🞎 Wood [Mbao]

🞎 Tiles [Vigae]

🞎 Cement [Sementi]

🞎 Dirt or mud [Vumbi/tope]

🞎 Dung [Kinyesi cha mifugo]

🞎 Other [Nyinginezo] [ ]

2.10 For the house that the head of the compound sleeps in, what is the floor made of [Kwa nyumba anayolala mkuu wa kaya, sakafu imetengenezwa na nini]?

🞎 Brick [Matofali]

🞎 Cement [Sementi]

🞎 Wood [Mbao]

🞎 Dirt or mud [Vumbi/tope]

🞎 Dung [Kinyesi cha mifugo]

🞎 Tile or linoleum [Vigae/sakafu ya mpira]

🞎 Other [Nyinginezo] [ ]

2.11 For the house that the head of the compound sleeps in, what are the walls made of [Kwa nyumba anayolala mkuu kaya, ukuta umetengenezwa na nini]?

🞎 Mud or manure [Tope au kinyesi cha mifugo]

🞎 Burnt bricks [Tofali zilizochomwa]

🞎 Mud bricks, uncooked [Tofali za matope]

🞎 Cement bricks [Sementi]

🞎 Wood [Mbao]

🞎 Stone [Mawe]

🞎 Thatch [Nyasi]

🞎 Other [Nyinginezo] [ ]

2.12 Do members of this compound own any of the following items [Je, wakazi wa kaya hii wanamiliki chochote kati ya vitu vifuatavyo]?

🞎 Ox plough [Chombo/kifaa]

🞎 Ox cart [Mkokoteni wa ng’ombe]

🞎 Bicycle [Baisikeli]

🞎 Motorbike [Pikipiki]

🞎 Car [Gari]

🞎 Tractor [Trekta]

🞎 Mobile phone [Simu ya mkononi]

🞎 Radio [Redio]

🞎 Television [Luninga]

🞎 Sofa [Makochi]

🞎 Refrigerator [Jokofu (Friji)]

**3. LAND AND CROPS**

3.1 Do members of this compound own land [Je, wanakaya wa kaya hii wanamliki ardhi]?

🞎 Yes 🞎 No

3.2 How much land do members of this compound own in total (in acres) [Ni kiasi gani cha ardhi wanakaya wa kaya hii wanamliki kwa ujumla (kwa eka)]? [ ]

3.3. Do members of the compound have a title for this land [Je, wanakaya wa kaya hii wana hati miliki ya hii ardhi]?

🞎 Yes 🞎 No

3.4 Where is the land title from [Je, hati miliki ya hii ardhi imetoka wapi]?

🞎 Local (traditional)

🞎 Government

🞎 Other [ ]

3.5 Were any crops grown by members of this compound during the last 12 months [Kuna mazao yeyote yalioteshwa na watu wa kaya hili katika miezi 12 iliyopita]?

🞎 Yes 🞎 No

3.6 Which crops did members of the compound grow in the past 12 months [Kwa miezi 12 iliyopita ni mazao gani yalioteshwa na watu wa kaya hili]?

🞎 Rice [Mpunga] 🞎 Millet [Uwele]

🞎 Sorghum [Mtama] 🞎 Maize [Mahindi]

🞎 Sesame [Sesame] 🞎 Cassava [Muhogo]

🞎 Sweet potato [Viazi vitamu] 🞎 Beans [Maharagwe]

🞎 Cabbage [Kabeji] 🞎 Lettuce [Saladi]

🞎 Tomato [Nyanya] 🞎 Banana [Ndizi]

🞎 Cotton [Pamba] 🞎 Coffee [Kahawa]

🞎 Potato [Viazi] 🞎 Avocado [Parachichi]

🞎 Spinach [Spinachi] 🞎 Sugarcane [Miwa] 🞎 Other [Nyinginezo]

3.7 How many years have members of this compound been growing crops [Ni kwa miaka mingapi watu wana kaya hii wameotesha mazao]? [ ]

3.8 Did this compound sell any crops in the last 12 months [Je, umeuza mazao yoyote katika miezi 12 iliyopita]?

🞎 Yes 🞎 No

**4. HOUSEHOLD LIVESTOCK OWNERSHIP**

4.1 Are any cattle currently kept at this compound [Je, kuna ng'ombe wowote wanafugwa kwenye boma hili kwa sasa]?

🞎 Yes 🞎 No

4.2 What is the total number of cattle that are managed together at this compound [Kuna jumla ya ng'ombe wangapi wanaotunzwa pamoja katika boma hili]? [ ]

4.3 Of these [NUMBER] cattle, how many are in the following breeds [Wangapi kati ya ngombe hawa]:

Indigenous [ ]

Crosses [ ]

Exotic [ ]

4.4 Of the [NUMBER] indigenous cattle, how many are the following types [Wangapi kati ya ngombe wakienyeji hawa]:

Adult male [ ]

Adult female [ ]

Juvenile male [ ]

Juvenile female [ ]

4.5 Of the [NUMBER] cross cattle, how many are the following types [Wangapi kati ya ngombe chotara hawa]:

Adult male [ ]

Adult female [ ]

Juvenile male [ ]

Juvenile female [ ]

4.6 Of the [NUMBER] exotic cattle, how many are the following types [Wangapi kati ya ngombe wa kisasa hawa]:

Adult male [ ]

Adult female [ ]

Juvenile male [ ]

Juvenile female [ ]

4.7 Are any goats currently kept at this compound [Je, kuna mbuzi wowote wanafugwa kwenye boma hili kwa sasa]?

🞎 Yes 🞎 No

4.8 What is the total number of goats that are managed together at this compound [Kuna jumla ya mbuzi wangapi wanaotunzwa pamoja katika boma hili]? [ ]

4.9 Of these [NUMBER] goats, how many are in the following breeds [Wangapi kati ya mbuzi hawa]:

Indigenous [ ]

Crosses [ ]

Exotic [ ]

4.10 Of the [NUMBER] indigenous goats, how many are the following types [Wangapi kati ya mbuzi wakienyeji hawa]:

Adult male [ ]

Adult female [ ]

Juvenile male [ ]

Juvenile female [ ]

4.11 Of the [NUMBER] cross goats, how many are the following types [Wangapi kati ya mbuzi chotara hawa]:

Adult male [ ]

Adult female [ ]

Juvenile male [ ]

Juvenile female [ ]

4.12 Of the [NUMBER] exotic goats, how many are the following types [Wangapi kati ya mbuzi wa kisasa hawa]:

Adult male [ ]

Adult female [ ]

Juvenile male [ ]

Juvenile female [ ]

4.13 Are any sheep currently kept at this compound [Je, kuna kondoo wowote wanafugwa kwenye boma hili kwa sasa]?

🞎 Yes 🞎 No

4.14 What is the total number of sheep that are managed together at this compound [Kuna jumla ya kondoo wangapi wanaotunzwa pamoja katika boma hili]? [ ]

4.15 Of these [NUMBER] sheep, how many are in the following breeds [Wangapi kati ya kondoo hawa]:

Indigenous [ ]

Crosses [ ]

Exotic [ ]

4.16 Of the [NUMBER] indigenous sheep, how many are the following types [Wangapi kati ya mbuzi wakienyeji hawa]:

Adult male [ ]

Adult female [ ]

Juvenile male [ ]

Juvenile female [ ]

4.17 Of these [NUMBER] cross sheep, how many are the following types [Wangapi kati ya kondoo chotara hawa]:

Adult male [ ]

Adult female [ ]

Juvenile male [ ]

Juvenile female [ ]

4.18 Of [NUMBER] exotic sheep, how many are the following types [ [Wangapi kati ya mbuzi wa kisasa hawa]::

Adult male [ ]

Adult female [ ]

Juvenile male [ ]

Juvenile female [ ]

**5. OTHER ANIMALS IN THE HOUSEHOLD**

5.1 Are any of the following animals owned in this compound [Kuna yoyote kati ya wanyama wafuatao wanamlikiwa katika boma hili]? [ ]

🞎 Dogs [Mbwa]

How many dogs are there [Ni mbwa wangapi wapo]? [ ]

🞎 Cats [Paka]?

How many cats are there [Ni paka wangapi wapo]? [ ]

🞎 Donkeys [Punda]?

How many donkeys are there [Ni punda wangapi wapo]? [ ]

🞎 Pigs [Nguruwe]?

How many pigs are there [Ni nguruwe wangapi wapo]? [ ]

🞎 Chickens [Kuku]?

How many chickens are there [Ni kuku wangapi wapo]? [ ]

🞎 Other birds [Ndege wengine]?

How many other birds are there [Ni ndege wengine wangapi wapo]? [ ]

🞎 Other animals [Wanyama wengine]?

What are these other animals [Ni wanyama gani hao]? [ ]

How many [NUMBER] (other animals) are there [Hao [NUMBER](wanyama wengine) wako wangapi]? [ ]

**6. LIVESTOCK INTRODUCTIONS AND LOSSES**

6.1 How many cattle were born in this compound in the past 12 months [Ng'ombe wangapi wamezaliwa katika boma hili katika miezi 12 iliyopita]? [ ]

6.2 Have any cattle been introduced into this compound in the past 12 months? [Kuna ng'ombe yeyote aliyeliletwa katika kundi lenu kwa kipindi cha miezi 12 iliyopita]

🞎 Yes 🞎 No

6.3 How many of the introduced cattle were purchased [Wangapi kati yao walionunuliwa]? [ ]

6.4 How many of the introduced cattle were not purchased (e.g. a gift, dowry, exchange etc) [Wangapi hawakununuliwa au waliletwa kama zawadi, mahari, kubadalishana]? [ ]

6.5 Where did you buy the [NUMBER] cattle introduced into your household's herd [Kwa hao ng'ombe [NUMBER] walionunuliwa walitokea wapi]?

🞎 Direct from another household

🞎 Direct from market

🞎 Livestock trader in this village (outside market)

🞎 Livestock trader in another village (outside market)

🞎 Don’t know

🞎 Other [Nyinginezo] [ ]

6.6 Have any cattle kept in this compound died in the past 12 months (not through slaughter) [Kuna ng'ombe yoyote anayefugwa kwenye boma lako amekufa katika miezi 12 iliyopita (siyo kwa kuchinjwa)]?

🞎 Yes 🞎 No

6.7 What did these animals die of [Hawa wanyama walikufa na nini]?

Specify other [Vinginevyo (ainisha)]:

🞎 Drought [Ukame]

🞎 Predation [Kuliwa na mnyama]

🞎 Disease [Ugonjwa]

🞎 Trauma [Kuumia]

🞎 Other [Nyinginezo] [ ]

🞎 Don’t know [Sijui]

6.8 How many died from disease [Wangapi walikufa kutokana na magonjwa]? [ ]

6.9 How many goats were born in this household in the past 12 months [mbuzi wangapi wamezaliwa katika boma hili katika miezi 12 iliyopita]? [ ]

6.10 Have any goats been introduced into this household in the past 12 months? [Kuna mbuzi yeyote aliyeliletwa katika kundi lenu kwa kipindi cha miezi 12 iliyopita]

🞎 Yes 🞎 No

6.11 How many of the introduced goats were purchased [Wangapi kati yao walionunuliwa]?

[ ]

6.12 How many of the introduced goats were not purchased (e.g. a gift, dowry, exchange etc) [Wangapi hawakununuliwa au waliletwa kama zawadi, mahari, kubadalishana]? [ ]

6.13 Where did you buy the [NUMBER] goats introduced into your household's herd [Kwa hao mbuzi [NUMBER] walionunuliwa walitokea wapi]?

🞎 Direct from another household

🞎 Direct from market

🞎 Livestock trader in this village (outside market)

🞎 Livestock trader in another village (outside market)

🞎 Don’t know

🞎 Other [Nyinginezo] [ ]

6.14 Have any goats kept by your household died in the past 12 months (not through slaughter) [Kuna mbuzi yoyote anayefugwa kwenye boma lako amekufa katika miezi 12 iliyopita (siyo kwa kuchinjwa)]?

🞎 Yes 🞎 No

6.15 What did these animals die of [Hawa wanyama walikufa na nini]?

🞎 Drought [Ukame]

🞎 Predation [Kuliwa na mnyama]

🞎 Disease [Ugonjwa]

🞎 Trauma [Kuumia]

🞎 Other [Nyinginezo] [ ]

🞎 Don’t know [Sijui]

6.16 How many died from disease [Wangapi walikufa kutokana na magonjwa]? [ ]

6.17 How many sheep were born in this household in the past 12 months [kondoo wangapi wamezaliwa katika boma hili katika miezi 12 iliyopita]? [ ]

6.18 Have any sheep been introduced into this household in the past 12 months? [Kuna kondoo yeyote aliyeliletwa katika kundi lenu kwa kipindi cha miezi 12 iliyopita]

🞎 Yes 🞎 No

6.19 How many of the introduced sheep were purchased [Wangapi kati yao walionunuliwa]?

[ ]

6.20 How many of the introduced sheep were not purchased (e.g. a gift, dowry, exchange etc) [Wangapi hawakununuliwa au waliletwa kama zawadi, mahari, kubadalishana]? [ ]

6.21 Where did you buy the [NUMBER] sheep introduced into your household's herd [Kwa hao kondoo [NUMBER] walionunuliwa walitokea wapi]?

🞎 Direct from another household

🞎 Direct from market

🞎 Livestock trader in this village (outside market)

🞎 Livestock trader in another village (outside market)

🞎 Don’t know

🞎 Other [ ]

6.22 Have any sheep kept by your household died in the past 12 months (not through slaughter) [Kuna kondoo yoyote anayefugwa kwenye boma lako amekufa katika miezi 12 iliyopita (siyo kwa kuchinjwa)]?

🞎 Yes 🞎 No

6.23 What did these animals die of [Hawa wanyama walikufa na nini]?

Specify other [Vinginevyo (ainisha)]:

🞎 Drought [Ukame]

🞎 Predation [Kuliwa na mnyama]

🞎 Disease [Ugonjwa]

🞎 Trauma [Kuumia]

🞎 Other [Nyinginezo] [ ]

🞎 Don’t know [Sijui]

6.24 How many died from disease [Wangapi walikufa kutokana na magonjwa]?  [ ]

**7. ANIMAL ILLNESS**

7.1 Have any animals in this compound been unwell (but not died) in the past 12 months?

🞎 Yes 🞎 No

Please tell us about the illness that affected the most cattle

7.2 How many cattle were affected [ ]

7.3 Which age group was affected?

🞎 Only adults

🞎 Only juveniles

🞎 Adults and juveniles

7.4 Which sex was affected?

🞎 Only females

🞎 Only males

🞎 Males and females

7.5 What signs did you observe?

🞎 Milk drop

🞎 Staring coat

🞎 Regurgitation

🞎 Diarrhoea

🞎 Nasal discharge

🞎 Coughing

🞎 Listlessness

🞎 Recumbancy

🞎 Abdominal pain

🞎 Jaundice

🞎 Inappetance

🞎 Lameness

🞎 Weight loss

🞎 Other [ ]

7.6 Do you know what the cause was?

🞎 Yes 🞎 No

7.7 What was the cause? [ ]

7.8 How many months ago did this occur? [ ]

7.9 Were there any other (different) episodes of illness in cattle?

🞎 Yes 🞎 No

7.10 How many cattle were affected? [ ]

7.11 Which age group was affected?

🞎 Only adults

🞎 Only juveniles

🞎 Adults and juveniles

7.12 Which sex was affected?

🞎 Only females

🞎 Only males

🞎 Males and females

7.13 What signs did you observe?

🞎 Milk drop

🞎 Staring coat

🞎 Regurgitation

🞎 Diarrhoea

🞎 Nasal discharge

🞎 Coughing

🞎 Listlessness

🞎 Recumbancy

🞎 Abdominal pain

🞎 Jaundice

🞎 Inappetance

🞎 Lameness

🞎 Weight loss

🞎 Other [ ]

7.14 Do you know what the cause was?

🞎 Yes 🞎 No

7.15 What was the cause? [ ]

7.16 How many months ago did this occur? [ ]

7.17 Were there any other (different) episodes of illness in cattle?

🞎 Yes 🞎 No

7.18 How many cattle were affected? [ ]

7.19 Which age group was affected?

🞎 Only adults

🞎 Only juveniles

🞎 Adults and juveniles

7.20 Which sex was affected?

🞎 Only females

🞎 Only males

🞎 Males and females

7.21 What signs did you observe?

🞎 Milk drop

🞎 Staring coat

🞎 Regurgitation

🞎 Diarrhoea

🞎 Nasal discharge

🞎 Coughing

🞎 Listlessness

🞎 Recumbancy

🞎 Abdominal pain

🞎 Jaundice

🞎 Inappetance

🞎 Lameness

🞎 Weight loss

🞎 Other [ ]

7.22 Do you know what the cause was?

🞎 Yes 🞎 No

7.23 What was the cause? [ ]

7.24 How many months ago did this occur? [ ]

Please tell us about the illness that affected the most goats

7.25 How many goats were affected? [ ]

7.26 Which age group was affected?

🞎 Only adults

🞎 Only juveniles

🞎 Adults and juveniles

7.27 Which sex was affected?

🞎 Only females

🞎 Only males

🞎 Males and females

7.28 What signs did you observe?

🞎 Milk drop

🞎 Staring coat

🞎 Regurgitation

🞎 Diarrhoea

🞎 Nasal discharge

🞎 Coughing

🞎 Listlessness

🞎 Recumbancy

🞎 Abdominal pain

🞎 Jaundice

🞎 Inappetance

🞎 Lameness

🞎 Weight loss

🞎 Other [ ]

7.29 Do you know what the cause was?

🞎 Yes 🞎 No

7.30 What was the cause? [ ]

7.31 How many months ago did this occur? [ ]

7.32 Were there any other (different) episodes of illness in goats?

🞎 Yes 🞎 No

7.33 How many goats were affected? [ ]

7.34 Which age group was affected?

🞎 Only adults

🞎 Only juveniles

🞎 Adults and juveniles

7.35 Which sex was affected?

🞎 Only females

🞎 Only males

🞎 Males and females

7.36 What signs did you observe?

🞎 Milk drop

🞎 Staring coat

🞎 Regurgitation

🞎 Diarrhoea

🞎 Nasal discharge

🞎 Coughing

🞎 Listlessness

🞎 Recumbancy

🞎 Abdominal pain

🞎 Jaundice

🞎 Inappetance

🞎 Lameness

🞎 Weight loss

🞎 Other [ ]

7.37 Do you know what the cause was?

🞎 Yes 🞎 No

7.38 What was the cause? [ ]

7.39 How many months ago did this occur? [ ]

7.40 Were there any other (different) episodes of illness in goats?

🞎 Yes 🞎 No

7.41 How many goats were affected? [ ]

7.42 Which age group was affected?

🞎 Only adults

🞎 Only juveniles

🞎 Adults and juveniles

7.43 Which sex was affected?

🞎 Only females

🞎 Only males

🞎 Males and females

7.44 What signs did you observe?

🞎 Milk drop

🞎 Staring coat

🞎 Regurgitation

🞎 Diarrhoea

🞎 Nasal discharge

🞎 Coughing

🞎 Listlessness

🞎 Recumbancy

🞎 Abdominal pain

🞎 Jaundice

🞎 Inappetance

🞎 Lameness

🞎 Weight loss

🞎 Other [ ]

7.45 Do you know what the cause was?

🞎 Yes 🞎 No

7.46 What was the cause? [ ]

7.47 How many months ago did this occur? [ ]

Please tell us about the illness that affected the most sheep

7.48 How many sheep were affected? [ ]

7.49 Which age group was affected?

🞎 Only adults

🞎 Only juveniles

🞎 Adults and juveniles

7.50 Which sex was affected?

🞎 Only females

🞎 Only males

🞎 Males and females

7.51 What signs did you observe?

🞎 Milk drop

🞎 Staring coat

🞎 Regurgitation

🞎 Diarrhoea

🞎 Nasal discharge

🞎 Coughing

🞎 Listlessness

🞎 Recumbancy

🞎 Abdominal pain

🞎 Jaundice

🞎 Inappetance

🞎 Lameness

🞎 Weight loss

🞎 Other [ ]

7.52 Do you know what the cause was?

🞎 Yes 🞎 No

7.53 What was the cause? [ ]

7.54 How many months ago did this occur? [ ]

7.55 Were there any other (different) episodes of illness in sheep?

🞎 Yes 🞎 No

7.56 How many sheep were affected? [ ]

7.57 Which age group was affected?

🞎 Only adults

🞎 Only juveniles

🞎 Adults and juveniles

7.58 Which sex was affected?

🞎 Only females

🞎 Only males

🞎 Males and females

7.59 What signs did you observe?

🞎 Milk drop

🞎 Staring coat

🞎 Regurgitation

🞎 Diarrhoea

🞎 Nasal discharge

🞎 Coughing

🞎 Listlessness

🞎 Recumbancy

🞎 Abdominal pain

🞎 Jaundice

🞎 Inappetance

🞎 Lameness

🞎 Weight loss

🞎 Other [ ]

7.60 Do you know what the cause was?

🞎 Yes 🞎 No

7.61 What was the cause? [ ]

7.62 How many months ago did this occur? [ ]

7.63 Were there any other (different) episodes of illness in sheep?

🞎 Yes 🞎 No

7.64 How many sheep were affected? [ ]

7.65 Which age group was affected?

🞎 Only adults

🞎 Only juveniles

🞎 Adults and juveniles

7.66 Which sex was affected?

🞎 Only females

🞎 Only males

🞎 Males and females

7.67 What signs did you observe?

🞎 Milk drop

🞎 Staring coat

🞎 Regurgitation

🞎 Diarrhoea

🞎 Nasal discharge

🞎 Coughing

🞎 Listlessness

🞎 Recumbancy

🞎 Abdominal pain

🞎 Jaundice

🞎 Inappetance

🞎 Lameness

🞎 Weight loss

🞎 Other [ ]

7.68 Do you know what the cause was?

🞎 Yes 🞎 No

7.69 What was the cause? [ ]

7.70 How many months ago did this occur? [ ]

7.71 Has milk production changed in any species in the past 12 months?

🞎 Cattle [Ng’ombe]

🞎 Goats [Mbuzi]

🞎 Sheep [Kondoo]

7.72 How has it changed in cattle?

🞎 Increased

🞎 Decreased

🞎 Don’t know

7.73 How has it changed in goats?

🞎 Increased

🞎 Decreased

🞎 Don’t know

7.74 How has it changed in sheep?

🞎 Increased

🞎 Decreased

🞎 Don’t know

7.75 Have any species had joint problems in the past 12 months?

🞎 Yes 🞎 No

**8. LIVESTOCK MANAGEMENT**

8.1 Have any animals in your herd been vaccinated in the past 24 months [Je, kuna wanyama katika kundi wamepata chanjo katika kipindi cha miezi 24 iliyopita]?

🞎 Yes 🞎 No

8.2. Against which diseases [Ni chanjo dhidi ya magonjwa gani]?

🞎 CBPP

🞎 Anthrax

🞎 FMD

🞎 LSD

🞎 RVF

🞎 Sheep pox

🞎 Goat pox

🞎 Other [ ]

8.3 How are cattle in this compound grazed during the wet and dry season [Unawalishaje ng'ombe wa boma hili kipindi cha mvua na kiangazi]?

Free ranging [Wanajichunga wenyewe]: 🞎 Dry 🞎 Wet 🞎 Neither

Herded [Wanachungwa]: 🞎 Dry 🞎 Wet 🞎 Neither

Tethered [Wanaofungwa]: 🞎 Dry 🞎 Wet 🞎 Neither

Zero grazed [Hawachungwi]: 🞎 Dry 🞎 Wet 🞎 Neither

8.4 How are goats in this compound grazed during the wet and dry season [Unawalishaje mbuzi wa boma hili kipindi cha mvua na kiangazi]?

Free ranging [Wanajichunga wenyewe]: 🞎 Dry 🞎 Wet 🞎 Neither

Herded [Wanachungwa]: 🞎 Dry 🞎 Wet 🞎 Neither

Tethered [Wanaofungwa]: 🞎 Dry 🞎 Wet 🞎 Neither

Zero grazed [Hawachungwi]: 🞎 Dry 🞎 Wet 🞎 Neither

8.5 How are sheep in this compound grazed during the wet and dry season [Unawalishaje kondoo wa boma hili kipindi cha mvua na kiangazi]?

Free ranging [Wanajichunga wenyewe]: 🞎 Dry 🞎 Wet 🞎 Neither

Herded [Wanachungwa]: 🞎 Dry 🞎 Wet 🞎 Neither

Tethered [Wanaofungwa]: 🞎 Dry 🞎 Wet 🞎 Neither

Zero grazed [Hawachungwi]: 🞎 Dry 🞎 Wet 🞎 Neither

8.6 Which of the following best describes the way you manage the herding of animals in this compound [Ipi kati yafuatayo inaeleza vizuri jinsi unavyotunza kundi la wanyama katika boma hili]:

🞎 Cattle, sheep and goats together [BO, OV, na CP pamoja]

🞎 Cattle separately, sheep and goats together [OV na CP pamoja]

🞎 Cattle with goats, sheep separately [BO na CP pamoja]

🞎 Cattle with sheep, goats separately [BO na OV pamoja]

🞎 All species separately [Kila aina inachungwa tofauti]

🞎 Other [Nyinginezo] [ ]

8.7 How long do your livestock walk (in minutes) from this compound for grazing on a normal day during the dry season? [Inachukuwa muda gani wa mifugo kutembea kwa ajili ya malisho (kwa dakika) kutoka boma hili wakati wa kiangazi]

Cattle [Ng’ombe] [ ]

Goats [Mbuzi] [ ]

Sheep [Kondoo] [ ]

8.9 How long do your livestock walk (in minutes) from this compound for grazing on a normal day during the wet season [[Inachukuwa muda gani wa mifugo kutembea kwa ajili ya malisho (kwa dakika) kutoka boma hili wakati wa mvua]?

Cattle [Ng’ombe] [ ]

Goats [Mbuzi] [ ]

Sheep [Kondoo] [ ]

8.10 How long do your livestock walk (in minutes) from this compound for water on a normal day during the dry season [Inachukuwa muda gani wa mifugo kutembea kwa ajili ya maji (kwa dakika) kutoka boma hili wakati wa kiangazi]?

Cattle [Ng’ombe] [ ]

Goats [Mbuzi] [ ]

Sheep [Kondoo] [ ]

8.11 How long do your livestock walk (in minutes) from this compound for water on a normal day during the wet season [Inachukuwa muda gani wa mifugo kutembea kwa ajili ya maji (kwa dakika) kutoka boma hili wakati wa masika]?

Cattle [Ng’ombe] [ ]

Goats [Mbuzi] [ ]

Sheep [Kondoo] [ ]

8.12 Are cattle from this compound regularly taken to seasonal camps for grazing [Je, ng'ombe wa boma hili wanapelekwa kwenye maboma ya muda kwa ajili ya malisho (ronjo)]?

🞎 Yes 🞎 No

8.13 Where are these seasonal camps (for cattle) [Haya maboma ya muda yapo wapi (kwa ng'ombe)]?

Village or place name [Kijiji au jina la sehemu] [ ]

Ward [Kata] [ ]

District [Wilaya] [ ]

8.14 Out of the [NUMBER] cattle in this compound, how many would normally go to seasonal grazing [Kati ya ng'ombe [NUMBER] ambao wako katika boma hii ni wangapi kwa kawaida wanaenda kwenye maboma ya muda kwa malisho]? [ ]

8.15 Are goats from this compound regularly taken to seasonal camps for grazing [Je, mbuzi wa boma hili wanapelekwa kwenye maboma ya muda kwa ajili ya malisho (ronjo)]?

🞎 Yes 🞎 No

8.16 Is this to the same place as for cattle [Sehemu hili ni sawa sawa za sehemu za n'gombe]?

🞎 Yes 🞎 No

8.17 Where are these seasonal camps (for goats)?

Village or place name [Kijiji au jina la sehemu] [ ]

Ward [Kata] [ ]

District [Wilaya] [ ]

8.18 Out of the [NUMBER] goats in this compound, how many would normally go to seasonal grazing [Kati ya mbuzi [NUMBER] ambao wako katika boma hli ni wangapi kwa kawaida wanaenda kwenye maboma ya muda kwa malisho]? [ ]

8.19 Are sheep from this compound regularly taken to seasonal camps for grazing [Je, kondoo wa boma hili wanapelekwa kwenye maboma ya muda kwa ajili ya malisho (ronjo)]?

🞎 Yes 🞎 No

8.20 Are these the same place as for cattle and goats [Sehemu hili ni sawa sawa za sehemu za n'gombe na mbuzi]?

🞎 Yes 🞎 No

8.21 Are these the same place as for cattle [Sehemu hili ni sawa sawa za sehemu za n'gombe]?

🞎 Yes 🞎 No

8.22 Are these the same place as for goats [Sehemu hili ni sawa sawa za sehemu za mbuzi]?

🞎 Yes 🞎 No

8.23 Where are these seasonal camps (for sheep) [Haya maboma ya muda yapo wapi (kwa kondoo)]?

Village or place name [Kijiji au jina la sehemu] [ ]

Ward [Kata] [ ]

District [Wilaya] [ ]

8.24 Out of the [NUMBER] sheep in this compound, how many would normally go to seasonal grazing [Kati ya kondoo [NUMBER] ambao wako katika boma hii ni wangapi kwa kawaida wanaenda kwenye maboma ya muda kwa malisho]? [ ]

8.25 Which of the following best describes the way you manage animals at night in this household [Ipi kati ya yafutatayo inaeleza vizuri jinsi unavyotunza wanyama wako katika kaya hii wakati wa usiku]:

🞎 Cattle, sheep and goats together [BO, OV, na CP pamoja]

🞎 Cattle separately, sheep and goats together [OV na CP pamoja]

🞎 Cattle with goats, sheep separately [BO na CP pamoja]

🞎 Cattle with sheep, goats separately [BO na OV pamoja]

🞎 All species separately [Kila aina inachungwa tofauti]

🞎 Other [Nyinginezo] [ ]

**9. BREEDING, PARTURITION, AND ABORTION**

9.1 How was service of livestock done in this compound in the past 12 months [Huduma ya upandishaji wa mifugo ilitolewa namna gani katika kundi lako kwa miezi 12 uliopita]?

Males from own herd: 🞎 Cattle 🞎 Goats 🞎 Sheep

[Madume (dume) kutoka kundi lako]

Males hired: 🞎 Cattle 🞎 Goats 🞎 Sheep

[Madume (dume) wakukodisha]

Males borrowed: 🞎 Cattle 🞎 Goats 🞎 Sheep

[Madume (dume) ya kuazima]

Males from other herds during grazing/watering: 🞎 Cattle 🞎 Goats 🞎 Sheep

[Wanaume kutoka wanyama wa wengine / kumwagilia]

wakati wa kulima

Artificial insemination: 🞎 Cattle 🞎 Goats 🞎 Sheep

[Uzalishaji kwa chupa]

Females taken elsewhere: 🞎 Cattle 🞎 Goats 🞎 Sheep

[Jike lilipelekwa pengine]

No service performed in past 12 months: 🞎 Cattle 🞎 Goats 🞎 Sheep

[Hakuna ushirikiano uliofanywa katika miezi 12

iliyopita]

Other [Nyinginezo]: 🞎 Cattle 🞎 Goats 🞎 Sheep

Species not owned or service not performed: 🞎 Cattle 🞎 Goats 🞎 Sheep

Specify other for cattle [ ]

Specify other for goats [ ]

Specify other for sheep [ ]

9.2 In the past 12 months, where were the young livestock owned by this compound born? [Je, indani wa miezi 12 iliyopita, mifugo wanao milikiwa na boma hili wamezaliwa wapi]?

In a (human) house in this compound : 🞎 Cattle 🞎 Goats 🞎 Sheep

In the compound (outside a house) : 🞎 Cattle 🞎 Goats 🞎 Sheep

Outside the compound in this village : 🞎 Cattle 🞎 Goats 🞎 Sheep

Outside the compound in a different village : 🞎 Cattle 🞎 Goats 🞎 Sheep

Other: 🞎 Cattle 🞎 Goats 🞎 Sheep

Species not owned/no births in past 12 months: 🞎 Cattle 🞎 Goats 🞎 Sheep

Specify other for cattle [ ]

Specify other for goats [ ]

Specify other for sheep [ ]

9.3 Do cattle that have just given birth usually get seperated from the main herd [Je, n'gombe ambao wamezaa wanatenganisha na kundi/wanyama wengine]?

🞎 Yes 🞎 No

9.4 For how long [Kwa muda gani]?

🞎 < 24 hours

🞎 12 to 24 hours

🞎 > 24 hours

9.5 How many days [Kwa siku ngapi]? [ ]

9.6 Do goats that have just given birth usually get seperated from the main herd [Je, mbuzi ambao wamezaa wanatenganisha na kundi/wanyama wengine]?

🞎 Yes 🞎 No

9.7 For how long [Kwa muda gani]?

🞎 < 24 hours

🞎 12 to 24 hours

🞎 > 24 hours

9.8 How many days [Kwa siku ngapi]? [ ]

9.9 Do sheep that have just given birth usually get seperated from the main herd [Je, kondoo ambao wamezaaa wanatenganisha na kundi/wanyama wengine]?

🞎 Yes 🞎 No

9.10 For how long [Kwa muda gani]?

🞎 < 24 hours

🞎 12 to 24 hours

🞎 > 24 hours

9.11 How many days [Kwa siku ngapi]? [ ]

9.12 What normally happens to the placenta and foetal membranes after one of your animals (sheep, cattle, or goats) gives birth [Kwa kawaida inakuwaje kwa kondo la nyuma baada ya mojawapo ya wanyama wako (ng'ombe, mbuzi, au kondoo) wanapozaa]?

🞎 Nothing, material left [Hakuna – viliachwa]

🞎 Eaten by family [Vililiwa na wakazi katika familia]

🞎 Buried [Vilifukiwa (zikwa)]

🞎 Burned [Vilichomwa]

🞎 Given to dogs [Vilipewa mbwa vikiwa vibichi

🞎 Thrown in bush [Vilitupwa porini]

🞎 Other [Nyinginezo] [ ]

9.13 How many cattle in this compound have aborted or delivered still born offspring in the past 12 months [Ni ng'ombe wangapi katika kaya yako wametupa mimba au kuzaa mtoto mfu katika miezi 12 iliyopita]? [ ]

9.14 How many goats in this compound have aborted or delivered still born offspring in the past 12 months [Ni mbuzi wangapi katika kaya yako wametupa mimba au kuzaa mtoto mfu katika miezi 12 iliyopita]? [ ]

9.15 How many sheep in this compound have aborted or delivered still born offspring in the past 12 months [Ni kondoo wangapi katika kaya yako wametupa mimba au kuzaa mtoto mfu katika miezi 12 iliyopita]? [ ]

9.16 What normally happens to the foetus, placenta and/or membranes after one of your livestock has an abortion or stillbirth [Kwa kawaida inakuwaje kwa kichanga, kondo la nyuma baada ya moja ya mifugo wako kutupa mimba au kuzaa mtoto mfu]?

🞎 Nothing, material left [Hakuna – viliachwa]

🞎 Eaten by family [Vililiwa na wakazi katika familia]

🞎 Buried [Vilifukiwa (zikwa)]

🞎 Burned [Vilichomwa]

🞎 Given to dogs [Vilipewa mbwa vikiwa vibichi

🞎 Thrown in bush [Vilitupwa porini]

🞎 Other [Nyinginezo] [ ]

9.17 What do you normally do with an animal after it has an abortion [Kwa kawaida unachukuwa hatua gani kwa mnyama baada ya kutupa mimba]?

Nothing : 🞎 Cattle 🞎 Goats 🞎 Sheep

Separate animal from herd for <24 hours : 🞎 Cattle 🞎 Goats 🞎 Sheep

Separate animal from herd for >24 hours : 🞎 Cattle 🞎 Goats 🞎 Sheep

Send animal for slaughter : 🞎 Cattle 🞎 Goats 🞎 Sheep

Sell animal : 🞎 Cattle 🞎 Goats 🞎 Sheep

Give animal away : 🞎 Cattle 🞎 Goats 🞎 Sheep

Get advice/treatment from outside household : 🞎 Cattle 🞎 Goats 🞎 Sheep

Treat the animal myself : 🞎 Cattle 🞎 Goats 🞎 Sheep

Other : 🞎 Cattle 🞎 Goats 🞎 Sheep

Never owned or had an abortion in this species : 🞎 Cattle 🞎 Goats 🞎 Sheep

Specify other for cattle [ ]

Specify other for goats [ ]

Specify other for sheep [ ]

9.18 Do you think you would be more likely to sell animals that have had an abortion than those that have not [Je, unafikiri kuna uwezekano mkubwa kuuza wanyama aliyetupa mimba kuliko wale ambao hawakutupa mimba]?

Cattle [Ng’ombe] [ ]

Goats [Mbuzi] [ ]

Sheep [Kondoo] [ ]

9.19 Do you think you would be more likely to slaughter animals that have had an abortion than those that have not [Je, unafikiri kuna uwezekano mkubwa kuchinja wanyama aliyetupa mimba kuliko wale ambao hawakutupa mimba]?

Cattle [Ng’ombe] [ ]

Goats [Mbuzi] [ ]

Sheep [Kondoo] [ ]

9.20 What treatment do you give when an animal has an abortion [Ni tiba gani gani unampa mnyama baada ya kutupa mimba]? [ ]

9.21 Who do you normally go to for advice/treament when an animal has an abortion [Ni nani kwa kawaida unakwenda kupata ushauri/matibabu kuhusu mimba ikitoka]?

🞎 Neighbour [Jirani]

🞎 Paravet [Paravet]

🞎 Veterinarian [Bwana mifugo]

🞎 Animal health assistant [Bwana afya msaidizi wa mifugo]

🞎 Agrovet [Bwana kilimo na mifugo]

🞎 Traditional healer [Mganga wa jadi]

🞎 Spiritual/faith healer [Imani/mponyaji wa imani]

🞎 Local livestock expert [Mtaalamu wa mifugo wa hapahapa]

🞎 Other [Nyinginezo] [ ]

9.22 Have you ever had a case of retained fetal membranes in livestock kept in this compound [Je, kulishawai kuwa na matukio ya kondo la nyuma kuacha kutoka katika mifugo inayotunzwa katika boma hili (ikihusisha kaya yako)]?

🞎 Yes 🞎 No

9.23 How many cattle in this compound have had a case of retained fetal membrane in the past 12 months [Ni ng'ombe wangapi wa kundi la kaya yako wamepata matukio ya kondo la nyuma kuacha kutoka katika miezi 12 iliyopita]? [ ]

9.24 How many goats in this compound have had a case of retained fetal membrane in the past 12 months [Ni miezi mingapi iliyopita mara ya mwisho kuwepo tukio la kondo la nyuma kuacha kutoka katika kundi la mbuzi wa kaya hii]? [ ]

9.25 How many sheep in this compund have had a case of retained fetal membrane in the past 12 months [Ni kondoo wangapi wa kundi la kaya yako wamepata matukio ya kondo la nyuma kuacha kutoka katika miezi 12 iliyopita]? [ ]

9.26 What actions do you take when an animal has a retained placenta [Je, unachukua hatua gani kama mnyama akishindwa kutoa kondo la nyuma]?

Nothing: 🞎 Cattle 🞎 Goats 🞎 Sheep

Separate animal from herd for <24 hours: 🞎 Cattle 🞎 Goats 🞎 Sheep

Separate animal from herd for >24 hours: 🞎 Cattle 🞎 Goats 🞎 Sheep

Send animal for slaughter: 🞎 Cattle 🞎 Goats 🞎 Sheep

Sell animal: 🞎 Cattle 🞎 Goats 🞎 Sheep

Give animal away: 🞎 Cattle 🞎 Goats 🞎 Sheep

Get advice/treatment from outside household: 🞎 Cattle 🞎 Goats 🞎 Sheep

Treat the animal myself: 🞎 Cattle 🞎 Goats 🞎 Sheep

Never owned/had retained fetal membrane: 🞎 Cattle 🞎 Goats 🞎 Sheep

Other [Nyinginezo] [ ]

9.27 Do you think you would be more likely to sell animals that have had a case of retained placenta than those that have not [Je, unafikiri kuna uwezekano mkubwa kuuza wanyama akishindwa kutoa kondo la nyuma kuliko wale ambao wametoa kondo la nyuma]?

Cattle [Ng’ombe] [ ]

Goats [Mbuzi] [ ]

Sheep [Kondoo] [ ]

9.28 Do you think you would be more likely to slaughter animals that have had a case of retained placenta than those that have not [Je, unafikiri kuna uwezekano mkubwa kuchinja wanyama akishindwa kutoa kondo la nyuma kuliko wale ambao wametoa kondo la nyuma]?

Cattle [Ng’ombe] [ ]

Goats [Mbuzi] [ ]

Sheep [Kondoo] [ ]

9.29 What treatment do you give when an animal in your herd has a retained placenta [Ni tiba gani unampa mnyama aliyeshindwa kutoa kondo la nyuma katika kundi lako]? [ ]

9.30 Who do you normally go to for advice/treament when your animal has a retained placenta [Kwa kawaida unakwenda kwa nani kupata ushauri/matibabu wakati mnyama wako ameshindwa kutoa kondo la nyuma]?

🞎 Neighbour [Jirani]

🞎 Paravet [Paravet]

🞎 Veterinarian [Bwana mifugo]

🞎 Animal health assistant [Bwana afya msaidizi wa mifugo]

🞎 Agrovet [Bwana kilimo na mifugo]

🞎 Traditional healer [Mganga wa jadi]

🞎 Spiritual/faith healer [Imani/mponyaji wa imani]

🞎 Local livestock expert [Mtaalamu wa mifugo wa hapahapa]

🞎 Other [Nyinginezo] [ ]

9.31 Have you identified infertile male or female livestock in this compound in the past 12 months [Je, umegunduwa mfugo wowote dume au jike amabye ni tasa katika boma hili miezi 12 iliyopita (ikihusisha boma lako)]?

🞎 Yes 🞎 No

9.32 How many male cattle have been identified as infertile in the past 12 months [Ni madume mangapi ya ng'ombe wa kundi la kaya yako wamegundulika kuwa tasa miezi 12 iliyopita]? [ ]

9.33 How many female cattle have been identified as infertile in the past 12 months [Ni majike mangapi ya ng'ombe wa kundi la kaya yako wamegundulika kuwa tasa miezi 12 iliyopita]? [ ]

9.34 How many male goats have been identified as infertile in the past 12 months [Ni madume mangapi ya mbuzi wa kundi la kaya yako wamegundulika kuwa tasa miezi 12 iliyopita]? [ ]

9.35 How many female goats have been identified as infertile in the past 12 months [Ni majike mangapi ya mbuzi wa kundi la kaya yako wamegundulika kuwa tasa miezi 12 iliyopita]? [ ]

9.36 How many male sheep have been identified as infertile in the past 12 months [Ni madume mangapi ya kondoo wa kundi la kaya yako wamegundulika kuwa tasa miezi 12 iliyopita]? [ ]

9.37 How many female have been identified as infertile in the past 12 months [Ni majike mangapi ya kondoo wa kundi la kaya yako wamegundulika kuwa tasa miezi 12 iliyopita]?

[ ]

9.38 What actions do you take when you discover an animal is infertile [Unachukua hatua gani kwa kawaida wakati mnyama ni tasa]?

Nothing : 🞎 Cattle 🞎 Goats 🞎 Sheep

Kinachofanyika-wanyama walioadhirika wanatunzwa kama wengine

Send animal for slaughter: 🞎 Cattle 🞎 Goats 🞎 Sheep

[Kuchinja]

Sell animal: 🞎 Cattle 🞎 Goats 🞎 Sheep

[Kuuza]

Give animal away: 🞎 Cattle 🞎 Goats 🞎 Sheep

Kupeanwa

Get advice/treatment from outside household: 🞎 Cattle 🞎 Goats 🞎 Sheep

Ushauri au matibabu yanatafutwa nje ya kaya

Treat the animal myself:

[Kutibu mnyama mwenyewe] 🞎 Cattle 🞎 Goats 🞎 SheepOthe

Never owned or had infertility in this species:

[Haijawahi kumiliki au kutokuwa na ujinga

katika aina hii]

🞎 Cattle 🞎 Goats 🞎 Sheep

Other [Nyinginezo] [ ]

Do you think you would be more likely to sell animals that you know are infertile than those that are not [Je, unafikiri kuna uwezekano mkubwa kuuza wanyama ambayo ni tasa kuliko wale ambao sio tasa]?

Cattle [Ng’ombe] [ ]

Goats [Mbuzi] [ ]

Sheep [Kondoo] [ ]

9.39 Do you think you would be more likely to slaughter animals that you know are infertile than those that are not [Je, unafikiri kuna uwezekano mkubwa kuchinja wanyama ambayo ni tasa kuliko wale ambao sio tasa]?

Cattle [Ng’ombe] [ ]

Goats [Mbuzi] [ ]

Sheep [Kondoo] [ ]

9.40 What treatment do you give when an animal in your herd is infertile [Ni tiba gani unampa mnyama katika kundi lako ambayo ni tasa]? [ ]

9.41 Who do you normally go to for advice/treament when an animal in your herd is infertile [Kwa kawaida unakwenda kwa nani kupata ushauri/matibabu wakati mnyama wako ni tasa]?

🞎 Neighbour [Jirani]

🞎 Paravet [Paravet]

🞎 Veterinarian [Bwana mifugo]

🞎 Animal health assistant [Bwana afya msaidizi wa mifugo]

🞎 Agrovet [Bwana kilimo na mifugo]

🞎 Traditional healer [Mganga wa jadi]

🞎 Spiritual/faith healer [Imani/mponyaji wa imani]

🞎 Local livestock expert [Mtaalamu wa mifugo wa hapahapa]

🞎 Other [Nyinginezo] [ ]

**SECTION 10: ANIMALS AROUND THE COMPOUND:**

We would now like to ask you a few questions about other animals that you may have seen around your compound, your village, or while grazing [Sasa hivi tunaweza kuuliza maswali kuhusu wanyama wengine walioko karibu na boma lako au ndani ya kijiji chako].

10.1 Have you seen rodents or evidence of rodents (e.g. faeces, urine, noises, rodent tracks, rodent damage) in or around your house in the past month [Je, umewahi kuwaona panya katika nyumba yako siku thelathini zilizopita]?

🞎 Yes 🞎 No

10.2 Do any members of this household do anything to control rodents [Je, yeyote wa wanakaya anafanya chochote kuzuia hawa panya]?

🞎 Yes 🞎 No

10.3 What type of rodent control do you use [Njia gani huwa unatumia]?

🞎 Mechanical [Kuwatega, kuwapiga (mfano, mitego)]

🞎 Chemical [Kutumia dawa/kemikali (mfano, sumu)]

🞎 Biological [Kutumia njia za kibiologia (mfano, kufuga paka)]

🞎 Other [Nyinginezo]

10.4 Do you see any of the following animals around your village during normal dry and wet seasons [Je, unaona wanyana wafuatao katika kijiji chako wakati wa kiangazi wa kawaida]?

Small monkeys [Karunguyeye] 🞎 Dry 🞎 Wet

Baboons [Kima] 🞎 Dry 🞎 Wet

Elephants [Tembo] 🞎 Dry 🞎 Wet

Zebra [Pundamilia] 🞎 Dry 🞎 Wet

Buffalo [Nyati / mbogo] 🞎 Dry 🞎 Wet

Antelope [Swala] 🞎 Dry 🞎 Wet

Giraffe [Twiga] 🞎 Dry 🞎 Wet

Wildebeest [Nyumbu] 🞎 Dry 🞎 Wet

Big cats (e.g. Lion, leopard) 🞎 Dry 🞎 Wet

Wild pigs (e.g bushpig, warthog) 🞎 Dry 🞎 Wet
