# Supplementary material for: Livestock abortion surveillance in Tanzania reveals disease priorities and importance of timely collection of vaginal swab samples for attribution
Source: eLife. 2024 Dec 16;13:RP95296. doi: 10.7554/eLife.95296 (PMC11649233; doi:10.7554/eLife.95296)
Supplement: Supplementary file 4. [file elife-95296-supp4.docx]

**Supplementary File 4:**
